# Gender Identity Milestones and Hormone Utilization in Transgender Men and Women in China

**DOI:** 10.1001/jamanetworkopen.2025.52440

**Published:** 2026-01-06

**Authors:** Jiayu Hou, Bailin Pan, Yinuo Chen, Yuanyuan Wang, Xu Chang, Tianpei Hong, Ye Liu

**Affiliations:** 1 Department of Endocrinology and Metabolism, State Key Laboratory of Female Fertility Promotion, Peking University Third Hospital, Beijing, China; 2 Department of Plastic Surgery, Peking University Third Hospital, Beijing, China; 3 Key Laboratory of Brain, Cognition, and Education Sciences, South China Normal University, Ministry of Education, Guangzhou, China; 4 School of Psychology, South China Normal University, Guangzhou, China; 5 China Center for Studies of Psychological Application, South China Normal University, Guangzhou, China; 6 Guangdong Key Laboratory of Mental Health and Cognitive Science, South China Normal University, Guangzhou, China

## Abstract

**Importance:**

Little is known about how gender identity develops and how it affects gender-affirming hormone therapy (GAHT) utilization among transgender people in China.

**Objective:**

To investigate gender identity development among Chinese transgender men (TM) and transgender women (TW) and to explore how identity-related factors are associated with GAHT utilization.

**Design, Setting, and Participants:**

This cross-sectional study is based on the latest Chinese Transgender Health Survey targeting the transgender population in China conducted from May to December in 2021. Data analysis was completed in December 2024. Participants were recruited online via snowball sampling.

**Main Outcomes and Measures:**

The primary outcomes are the timing of gender identity development milestones (first perception of gender incongruence, confirmation, disclosure, and initiating GAHT) and GAHT utilization status. Multivariable binary logistic regression identified factors associated with GAHT-related behaviors and feedback.

**Results:**

A total of 4296 transgender people (1462 TM [34.0%] and 2834 TW [66.0%]; median [IQR] age, 21 [18-24] years) were included in the final analysis. The age distribution of first perceived gender incongruence exhibited a bimodal pattern, with peak occurrences at ages 5 to 6 and at 12 years. The median (IQR) ages at gender identity development milestones were all younger for TM than for TW: 6 (4-10) years vs 9 (6-12) years for perception, 14 (11-16) years vs 15 (12-17) years for confirmation, and 16 (14-19) years vs 17 (15-20) years for disclosure, whereas the age of initiating GAHT was older in TM than in TW (median [IQR] age, 19 [17-22] years vs 18 [16-21] years). The demand for GAHT (3759 participants [87.5%]), usage (2247 participants [52.3%]), and the rate of valid prescriptions among hormone users (339 participants [15.1%]) have all increased significantly compared with 2017. Being a TW and without a college education were associated with starting GAHT before age 16 years, while being a TM, having family disclosure of gender identity, and having official prescriptions were associated with positive feedback on GAHT.

**Conclusions and Relevance:**

In this cross-sectional study of Chinese TM and TW, gender identity development differed by gender, with TM recognizing incongruence earlier and TW progressing faster in initiating GAHT. Both groups showed strong demand for GAHT. Despite recent improvements in GAHT service accessibility, challenges persist in medical accessibility. These findings highlight the need for personalized support for transgender youths and underscore the importance of improving formal transgender health care services in China to enhance the well-being of this population.

## Key Points

**Question:**

What are the characteristics of gender identity development and gender-affirming hormone therapy (GAHT) utilization among Chinese transgender men and women?

**Findings:**

This cross-sectional study of 1462 transgender men and 2834 transgender women found that while transgender men began gender identity explorations earlier, transgender women progressed faster toward medical affirmation. Although access to qualified GAHT services has improved in China, challenges persist in medical accessibility and social acceptance.

**Meaning:**

These findings emphasize the need for early, individualized care for transgender youths and underscore the importance of expanding accessible and patient-centered transgender health care services in China.

## Teaser

This cross-sectional study investigates gender identity development and its association with use of gender-affirming hormone therapy among transgender individuals in China.

## Introduction

The term *transgender and gender diverse* (TGD) refers to individuals whose gender identities or expressions differ from their sex assigned at birth.^1^ Recent literature has reported an increasing TGD population, with prevalence estimates ranging from 17 to 33 per 100 000 according to diagnostic codes, or 0.3% to 2.7% depending on self-reported evidence; the rates are higher among children and adolescents than adults.^2^ Current evidence conceptualizes gender identity development as a biopsychosocial process shaped by biological, cognitive, and social factors within specific cultural and historical contexts.^3^ Literature suggests that gender incongruence typically emerges in early childhood, intensifies around the onset of puberty, and continues to evolve through adolescence into adulthood, although the timing and expression of identity formation vary across individuals and cultural settings.^4^ However, evidence remains limited regarding when a transgender person perceives, confirms, and discloses gender identity, especially in East Asian populations.

From a medical perspective, some transgender individuals experience gender dysphoria severe enough to require professional medical intervention, including gender-affirming hormone therapy (GAHT) and surgery. Appropriate GAHT within supportive health care systems has been demonstrated to be safe and impactful in reducing psychological distress and improving overall well-being.^1,5,6,7,8,9,10^ In line with international calls for equitable access to gender-affirming care,^11^ ensuring evidence-based, inclusive medical support is essential for the TGD population. The first national cross-sectional Chinese Transgender Health Survey conducted in 2017 revealed that lack of access to gender-affirming medical assistance in China was associated with high-risk behaviors, such as illegal hormone purchases and unsupervised hormone use.^12^ Since then, persistent efforts have been made in China to improve accessibility and standardization of gender-affirming medical assistance through the establishment of official regulations, gender clinics, and expert consensus on the diagnosis and treatments of gender incongruence.^13,14^ Given these advances, we expected that there would have been an increase in GAHT accessibility and utilization in recent years.

In this article, we analyzed data from the 2021 second national Chinese Transgender Health Survey to explore gender identity developmental patterns of Chinese transgender men (TM) and transgender women (TW), and to evaluate current GAHT utilization compared with 2017 data. These findings provide critical evidence for guardians, educators, health care practitioners, and policymakers to develop support systems and optimize medical services for this population.

## Methods

### Study Design

This cross-sectional study used data from the 2021 national Chinese Transgender Health Survey, a national cross-sectional survey conducted from May 6 to December 26, 2021, targeting the TGD population in China.^15^ It covered key areas such as demographics, socioeconomic status, gender identity development, family support, mental health, and gender-affirming medical and surgical care. In this study, we focus on the questions about gender identity development and GAHT. The 2021 survey was approved by the ethics committee of The Second Xiangya Hospital of Central South University, and was preregistered on the Open Science Framework.^16^ This post hoc analysis was approved by the ethics committee of Peking University Third Hospital. Since China currently lacks established medical guidelines for GAHT targeting gender nonbinary individuals, this group of participants was excluded, and only participants identifying as TM and TW were included in the present analysis. This report adhered to the Strengthening the Reporting of Observational Studies in Epidemiology (STROBE) reporting guidelines for cross-sectional studies.^17^

### Participants

Participants were recruited nationwide through a snowball sampling strategy. The initial recruitment was conducted through TGD community organizations and our gender clinic in Beijing, and participants were encouraged to share the link on major Chinese social media platforms (eMethods 1 in Supplement 1). All participants provided electronic informed consent and were informed of their right to withdraw at any time. They completed the questionnaire in Chinese anonymously online. Inclusion criteria were (1) self-identification as TM or TW based on the 2-step gender identity measure; (2) aged at least 12 years at the time of survey completion; and (3) passing at least 2 of the 3 attention filter questions. Exclusion criteria were (1) response without informed consent; (2) duplicated questionnaires based on internet protocol address; (3) self-identification as cisgender, nonbinary, cross-dressing individual, or additional gender identities; and (4) being raised or current residency abroad or in the Taiwan region. Participants were informed of the study purpose and anonymity to reduce social desirability and recall bias. Attention checks and predefined inclusion criteria minimized measurement error. Recruitment through multiple community networks improved representativeness. Based on the results of the 2017 survey,^12^ the sample size for the 2021 survey was preset to be at least 5500.

### Defining Gender Identity

Gender identities were assessed using a 2-step approach, considered the gold standard in survey research.^18^ Sex assigned at birth was measured by asking each participant, “Which sex were you assigned at birth?” Response options included male, female, and other (to be filled by participants). Gender identity was measured by asking each participant, “If only one item can be chosen, which of the following is a better description of you?” Response options included man, woman, transgender man, transgender woman, nonbinary or genderqueer, cross-dressing individual, questioning, and none of the above. Participants assigned female at birth with a man or transgender man identity were defined as TM, and those assigned male at birth with a woman or transgender woman identity were defined as TW. See the eMethods 2 in Supplement 1 for further details of the 2021 survey and translation of items used in this study.

### Statistical Analysis

Quantitative variables are shown as medians and IQRs, and categorical variables are shown as counts and percentages. Group differences were tested with Mann-Whitney *U* test and Pearson χ^2^ test. Multivariable binary logistic regression was used to identify factors associated with GAHT-related behaviors and feedback and to generate odds ratios (ORs) and 95% CIs. Analyses were performed with SPSS Statistics software version 26.0 (IBM). Two-sided *P* < .05 was considered statistically significant.

## Results

**Figure 1.**
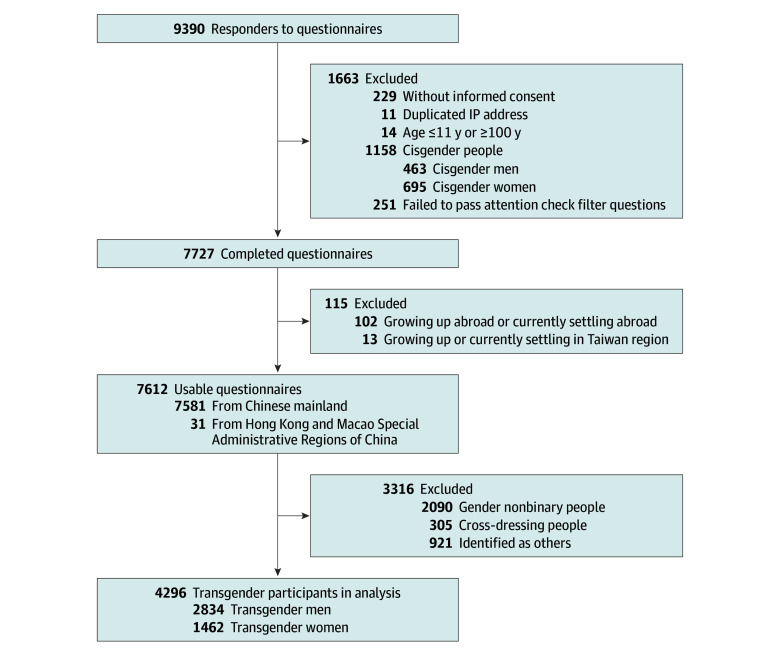
Flow Diagram of the Participants Abbreviation: IP, internet protocol.

Of 9390 responses, 7727 (82.3%) were considered complete. Since this study focuses on TM and TW in Chinese mainland, Hong Kong, and Macao, 2090 individuals identifying as gender nonbinary, 305 identifying as cross-dressing individuals, 921 identifying as other, and 115 individuals residing abroad or in the Taiwan region were excluded. Thus, a total of 4296 responses were completed by 1462 TM (34.0%) and 2834 TW (66.0%) (Figure 1). Their median (IQR) age was 21 (18-24) years, including 790 teenagers (18.4%) and 3506 adults (81.6%) aged 18 to 59 years. Most responders (2605 individuals [60.6%]) lived in big cities in China, and most (2845 responders [66.2%]) had education backgrounds of undergraduate degree or above (Table 1).

**Table 1.** Characteristics of Transgender Men and Women in 2021 Chinese Transgender Health Survey

Characteristic	Transgender individuals, No. (%)		
	Total (N = 4296)	Men (n = 1462)	Women (n = 2834)
Age, y			
Median (IQR)	21 (18-24)	21 (18-25)	20 (18-24)
12-17	790 (18.4)	280 (19.2)	510 (18.0)
18-59	3506 (81.6)	1182 (80.8)	2324 (82.0)
Ethnicity			
Han	4000 (93.1)	1343 (91.9)	2657 (93.8)
Other^a^	296 (6.9)	119 (8.1)	177 (6.2)
Current residence			
Big cities^b^	2605 (60.6)	915 (62.6)	1690 (59.6)
Other areas	1691 (39.4)	547 (37.4)	1144 (40.4)
Education background			
Less than high school	334 (7.8)	124 (8.5)	210 (7.4)
High school or technical secondary school	1117 (26.0)	352 (24.1)	765 (27.0)
Undergraduate	2553 (59.4)	857 (58.6)	1696 (59.8)
Bachelor’s degree	249 (5.8)	115 (7.9)	134 (4.7)
Master’s degree or higher	43 (1.0)	14 (1.0)	29 (1.0)

^a^ Other included participants of additional ethnicities including Hui, Manchu, Mongol, Tujia, Zhuang, and more.

^b^ Refers to Beijing, Shanghai, Shenzhen, and other provincial capital cities of China.

### Development of Gender Identity

**Figure 2.**
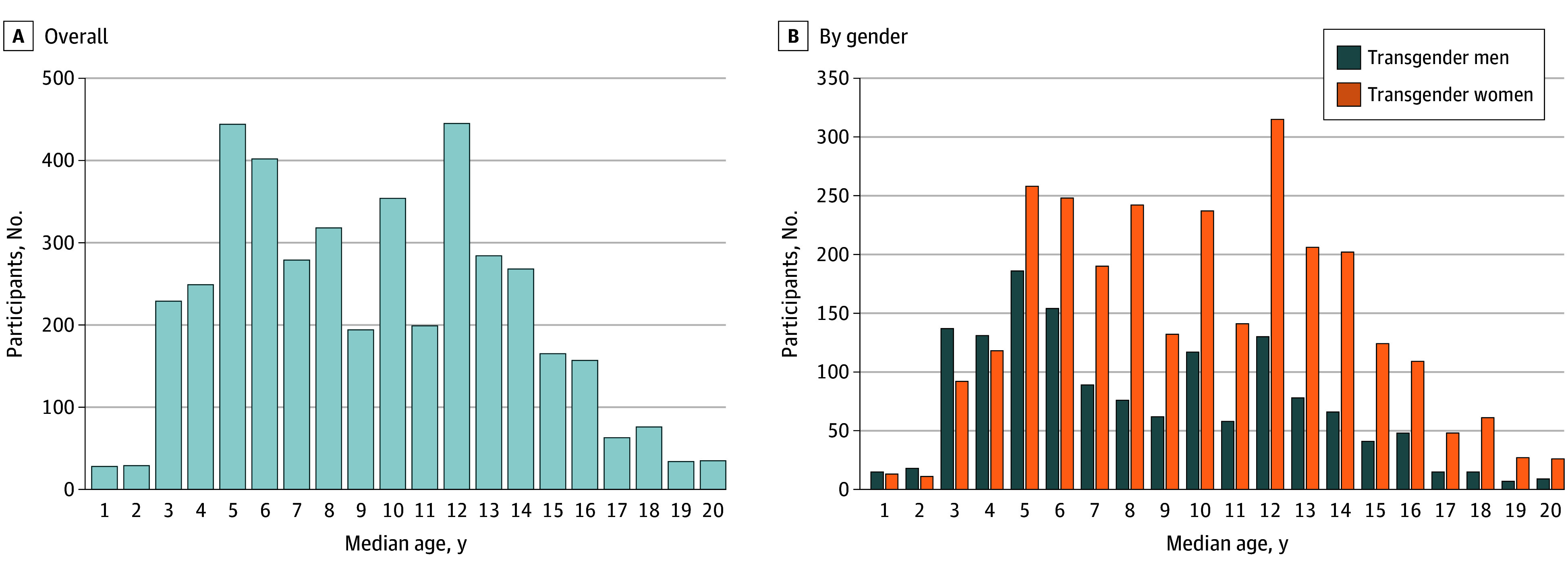
Age of First Perceiving Gender Incongruence for Chinese Transgender People A, Graph shows reported age for first perceiving gender incongruence among all Chinese transgender individuals surveyed. B, Graph shows reported age for first perceiving gender incongruence by gender. Individuals who reported an age older than 20 years (45 of 4297) are not shown.

Participants reported the age of first perception, confirmation, and disclosure of gender identity. Overall, the median (IQR) age of first perception of gender incongruence was 9 (6-13) years, showing 2 peaks at 5 to 6 years and at 12 years (Figure 2A). Similar trends presented among TM and TW (Figure 2B), but with a younger median (IQR) age for TM than for TW (7 [5-12] years vs 10 [6-13] years; *z* = –11.74; *P* < .001). The median (IQR) age of identity confirmation was 15 (12- 17) years, while 4.2% (183 respondents) reported that they were not sure about the age of identity confirmation. Similarly, the median (IQR) age of confirming gender identity was younger among TM than among TW (14 [12-16] years vs 15 [12- 18] years; *z* = –6.89; *P* < .001). The median (IQR) age for first telling others about gender identity was 16 (14-19) years for TM and 17 (15-20) years for TW (*z* = –8.34; *P* < .001). A total of 621 respondents (14.4%) reported having not yet told their gender identity to others. This rate was significantly higher among TW than TM (443 TW [15.6%] vs 178 TM [12.2%]; χ^2^ = 9.3, *P* = .002). The percentage of family awareness of gender identity (2754 [64.1%]) was higher than what was reported in 2017 (50.0%),^12^ while 1542 transgender people (35.9%) still reported that their family members did not know about their gender identity yet.

**Figure 3.**
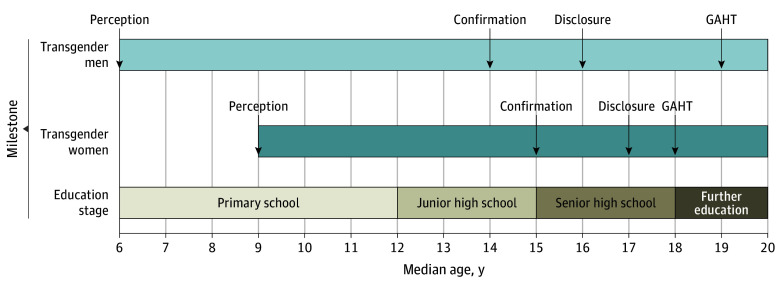
Gender Identity Development Pattern of Chinese Transgender People Gender identity development trajectories are shown by the median ages among 385 transgender men and 1622 transgender women who reported experiencing all 4 milestones of gender identity development: first perception, confirmation, disclosure, and initiating gender-affirming hormone therapy (GAHT). The timeline was compared with China’s compulsory education stages for reference.

For further insight, we studied 385 TM and 1622 TW who reported all of the following 4 important gender-related events, including perception, confirmation, and disclosure of gender incongruence, as well as initiation of GAHT. Median ages for these milestones are shown in Figure 3, with Chinese compulsory education arrangement as reference. TM reported younger median (IQR) ages compared with TW for perception (6 [4-10] years vs 9 [6-12] years; *z* = –9.29; *P* < .001), confirmation (14 [11- 16] years vs 15 [12-17] years; *z* = –4.42; *P* < .001), and disclosure (16 [14-19] years vs 17 [15-20] years; *z* = –4.75; *P* < .001) of gender incongruence, whereas TW initiated GAHT at a younger age (median [IQR], 18 [16-21] years vs 19 [17-22] years; *z* = –5.56; *P* < .001). Such patterns suggest that although TM tended to start their gender identity development earlier, TW were more proactive in seeking medical affirmation. Individuals who had completed all 4 stages reported significantly earlier perception of gender incongruence compared with those who had not disclosed their identity or initiated GAHT, both among TM (median [IQR] age, 6 [4-10] years vs 8 [5-12] years; *z* = –5.75; *P* < .001) and TW (median [IQR] age, 9 [6-12] years vs 11 [7-14] years; *z* = –8.69; *P* < .001).

### GAHT Utilization and Access

Next, we examined the utilization status of GAHT among Chinese TM and TW and investigated its association with gender identity milestones and other potential factors. As seen in Table 2, 3759 participants (87.5%) reported desiring GAHT, significantly higher than the 1036 participants (79.4%) in the 2017 survey (χ^2^ = 52.7, *P* < .001).^12^ TW were more likely than TM to express an intention to receive GAHT (2622 [92.5%] vs 1137 [77.8%]; χ^2^ = 191.8, *P* < .001). Among 2275 individuals who reported having once taken hormones, 28 reports of GAHT initiation prior to age 6 years were excluded due to implausibility, given the lack of medical indication and the practical and financial improbability of a young child obtaining medication independently. The remaining 2247 hormone ever-users (52.3%) were included in the following analysis. Among them, 1980 (88.1%) reported current hormone use. Consistent with GAHT demand, hormone history was more common among TW than TM (1847 TW [65.2%] vs 400 TM [27.4%]; χ^2^ = 552.8, *P* < .001).

**Table 2.** Desire and Usage of GAHT Among Transgender Individuals in China

GAHT behaviors	Participants, No. (%), by survey year		*P* value^b^	Participants, No. (%), by gender		*P* value^c^
	2021 (n = 4296)	2017 (n = 1304)^a^		Men (n = 1462)	Women (n = 2834)	
Desire to take GAHT	3759 (87.5)	1036 (79.4)	<.001	1137 (77.8)	2622 (92.5)	<.001
Current or previous GAHT	2247 (52.3)	602 (46.2)	<.001	400 (27.4)	1847 (65.2)	<.001
Obtaining hormone with valid prescription^d^	339 (15.1)	50 (8.3)	<.001	42 (10.5)	297 (16.1)	.005

Abbreviation: GAHT, gender-affirming hormone therapy.

^a^ Data are adapted from our previous research.^12^

^b^ *P* values are for 2021 survey vs 2017 survey.^12^

^c^ *P* values are for transgender men vs transgender women in 2021 survey.

^d^ Evaluated among current or previous hormone users.

There were 313 participants (14.0%) reporting first hormone use before age 16 years, which was younger than the usually recommended age limit for GAHT.^1,8,13^ Of the 2247 individuals with hormone use history, 339 (15.1%) obtained hormone medicines with prescription from certificated physicians, a rate significantly higher among TW than TM (297 TW [16.1%] vs 42 TM [10.5%]; χ^2^ = 8.0, *P* = .005). This rate was 8.3% (50 respondents) in the 2017 study, indicating a significant increase (χ^2^ = 18.5, *P* < .001) regarding the access to qualified GAHT service in China. The top 3 reasons for self-prescription were complicated procedures (1497 individuals [78.5%]), not knowing where to get a physician’s prescription (802 individuals [42.0%]), and financial constraints (769 individuals [40.3%]).

### Gender Identity Milestones and Other Potential Factors Associated With GAHT Utilization Behaviors

Multivariable binary logistic regression showed that being a TW (OR, 1.96; 95% CI, 1.32-2.91; *P* = .001) and having a below-college education (OR, 7.75; 95% CI, 5.92-10.20; *P* < .001) were associated with underage hormone use, whereas early perception (OR, 1.27; 95% CI, 0.98-1.64; *P* = .07) and family disclosure (OR, 1.35; 95% CI, 0.99-1.80; *P* = .06) were not statistically significantly associated with such use (eFigure 1 in Supplement 1). Being a TW (OR, 0.54; 95% CI, 0.38-0.76; *P* < .001), family disclosure (OR, 0.47; 95% CI, 0.34-0.65; *P* < .001), and having an undergraduate or above education (OR, 0.41; 95% CI, 0.31-0.55; *P* < .001) were associated with lower risks of obtaining hormones without medical prescription, whereas early realization of gender incongruence showed no significant association (OR, 0.92; 95% CI, 0.72-1.16; *P* = .47) (eFigure 2 in Supplement 1).

Participants were asked to give GAHT feedback using a single-item question. Among 2247 individuals with hormone history, 1670 (74.3%) reported improved academic or occupational performance, 252 (11.2%) gave neutral feedback, and 325 (14.5%) reported negative impacts. A multivariable binary logistic regression identified factors associated with positive GAHT feedback (vs neutral or negative). Being a TM (OR, 3.45; 95% CI, 2.44-4.76; *P* < .001), family disclosure (OR, 1.82; 95% CI, 1.47-2.25; *P* < .001), and official prescription (OR, 1.56; 95% CI, 1.16-2.10; *P* = .004) were significantly associated with positive feedback, while early perception (OR, 0.98; 95% CI, 0.80-1.20; *P* = .85) and underage hormone use (OR, 1.25; 95% CI, 0.92-1.68; *P* = .15) were not significantly associated with positive feedback (eFigure 3 in Supplement 1).

Of the 325 individuals giving negative feedback, 21 (6.5%) reported regretting hormone usage. The leading reason of regret was being unable to meet expected body change (15 individuals [83.0%]). Other reasons included hormone-related adverse events, no alleviation of gender anxiety, cost exceeding financial capacity, and family opposition (eTable in Supplement 1).

## Discussion

This cross-sectional study, which is based on the largest and most recent survey of TGD people in China, provides a comprehensive overview of gender identity development and GAHT status among Chinese TM and TW. Distinct patterns were observed between TM and TW in both identity development and GAHT utilization. Compared with 2017 data, participants demonstrated increased medical needs and improved access to qualified GAHT services, although overall utilization of formal transgender health care remained limited.

Two peaks were observed in the age of first perceived gender incongruence among our participants, 1 at age 5 to 6 years and 1 at 12 years. Developmental psychology suggests that gender identity stabilizes around age 3 to 5 years, while stereotype rigidity peaks at age 5 to 6 years before becoming more flexible thereafter.^19,20^ Therefore, the first peak may reflect the conflict between inner identity and socially reinforced stereotypes among peers. Such conflict is shaped by social context where family acceptance protects against adverse psychosocial outcomes, whereas an unsupportive school environment marked by bullying and gender-based harassment often exacerbates distress.^21,22^ The second peak overlaps with the onset of puberty, a crucial period when physical puberty, shifting social environments, and the discovery of sexuality may intensify or mitigate gender discomfort.^23,24^ The internet also plays a growing role as a cultural and information resource, offering access to knowledge and social affirmation often lacking in family or school contexts.^4,25,26,27^

A delay between first perceiving and disclosing gender identity was found, consistent with our 2017 survey,^12^ and the 2015 US Transgender Survey, where most transgender youths came out years after first recognizing incongruence.^28^ Our results also showed that more than 35% of transgender individuals remained silent to their families. Literature suggests that coming out for transgender people is a socially situated, ongoing process, where factors such as gender expectation from others, reflected confirmation, violence,^29^ and stigma^4^ are carefully assessed when deciding whether they choose to come out in a certain social situation. At home, transgender people might face verbal or physical violence, even identity conversion practice from their families, which is closely related with severe mental health problems.^15^ However, family awareness has increased markedly since 2017 (64.1% vs 50.0%), likely related to rising social visibility of gender diversity, greater public understanding, and official regulations of transgender health care in China.

We also found that TM tended to start gender identity exploration earlier but TW processed faster toward medical affirmation. This pattern might be affected by cultural expectations that stigmatize femininity in assigned boys but not vice versa for assigned girls. Interestingly, participants who completed all 4 identity-related milestones reported first perceiving gender incongruence at a significantly younger age, suggesting earlier realization may promote proactive identity exploration and medical affirmation. It is in line with past literature suggesting that children with an early realization of gender incongruence are prone to disclose gender identity and seek gender-affirming medical assistance sooner.^30,31^ These findings underscore the need for educators, parents, and health care practitioners to provide gender-sensitive education and early counseling, particularly for children and adolescents at critical developmental stages. Additionally, since TM reported younger age of gender-related events and were prone to purchase hormones without official prescription, gender identity–related consultation should start earlier in young TM to mitigate unsafe medical practices.

A substantial increase was observed in both the desire for and actual use of GAHT compared with 2017,^12^ reflecting growing awareness and medical accessibility. While it reflects the improvements in both public awareness of and accessibility to transgender medical care in China, only 15.1% of hormone users obtained medications with official prescriptions due to financial barriers, procedural complexity, or unawareness of available medical services. These data reveal persistent structural inequities regarding gender-affirming care resources in China, as qualified gender clinics are mainly located in first-tier cities. Furthermore, since 60.6% of participants in this study resided in big cities, we could expect even greater shortages among the general Chinese transgender population, especially in remote areas. More health care workers should be trained to provide professional and accessible care for transgender individuals in need. It is noteworthy that our sampling method may have led to a higher reported intention for gender-affirming care compared with other recent studies.^32,33^ This is because our snowball sampling initiated partly from visitors to our gender clinic, and that our sample contained a higher proportion of TW.

Moreover, 14.0% of respondents reported initiating hormone use before the age of 16 years, a practice generally discouraged by most existing GAHT guidelines. Being a TW and having an educational background below the undergraduate level were significantly associated with underage hormone use. This higher likelihood of underage hormone use among TW may reflect heightened gender dysphoria, while the association with lower education suggests disparities in access to reliable medical information, leading some individuals to take risky behaviors.

Despite the high rate of self-prescribed hormones, more individuals reported positive feedback. Multivariable analysis revealed that being a TM, family disclosure of identity, and obtaining prescribed hormones were significantly associated with positive GAHT feedback. These findings align with previous literature indicating high satisfaction and adherence to hormone therapy among transgender individuals, especially for those starting qualified therapy under supervision of a physician and supported by family.^34,35^

Collectively, our findings suggest a close link between gender identity development and medical affirmation behaviors. Family support and higher education appear to facilitate healthier medical decision-making and more positive treatment outcomes. Future efforts should focus on expanding access to qualified gender-affirming services beyond major cities, training health care professionals, and integrating online consultation and follow-up systems, which are already adopted in our center, to enhance accessibility and continuity of care nationwide.

### Limitations

This study has several limitations. First, the use of snowball sampling may have introduced selection bias related to regional distribution, socioeconomic status, and educational background. Second, our analysis was limited to participants currently identifying as TM and TW, which might oversimplify gender diversity. Although nonbinary identities were recognized, they were excluded from analysis due to the lack of standardized GAHT protocols for this group in China. Their data will be analyzed separately in future studies. In particular, individuals who had detransitioned and, thus, no longer identified as transgender may have been omitted, which remains a major concern in gender-affirming medical care research. Third, gender identity milestones were retrospectively recalled, which may introduce recall bias and fail to capture the multidimensional nature of gender identity formation. Another crucial limitation of this study is that our assessment of gender identity development and GAHT feedback relied on single-item questions, which cannot fully capture the complex domains such as gender congruence, psychological well-being, and life satisfaction. Systematic use of patient-reported outcome measures can enhance communication, improve care quality, and enable cross-study comparisons.^36,37,38^ Future surveys should adopt rigorously validated, comprehensive, and conflict-of-interest-free measures, such as the Gender Congruence and Life Satisfaction Scale^39^ and the Chinese version of the Gender Identity/Gender Dysphoria Questionnaire for Adolescents and Adults.^40^ To address these limitations, we are initiating a nationwide telephone-based epidemiological study supported by the National Social Science Fund of China to further characterize the diversity and longitudinal trends of TGD individuals. Future research will also incorporate validated, multidimensional patient-reported outcome measures to comprehensively evaluate treatment outcomes.

## Conclusions

In this cross-sectional study of TM and TW in China, we analyzed gender identity development patterns and GAHT utilization based on the largest and latest national survey targeting the Chinese TGD population. Gender identity development varied between genders, with TM reporting earlier awareness of incongruence, while TW exhibiting faster progression in transition. Both groups showed strong demand for GAHT. Although access to formal GAHT services has improved in recent years, challenges persist regarding family disclosure and medical accessibility. These findings highlight the need for inclusive public policies and health care systems that integrate education, family support, and accessible gender-affirming medical services to enhance the well-being of transgender people in China.

## Notes

Supplement 1. eMethods 1. 2021 Chinese Transgender Health Survey Recruitment eMethods 2. Questions in the 2021 Survey Questionnaire Relevant to this Study eMethods 3. Attention-check Questions in the 2021 Survey eFigure 1. Factors Associated with Gender-Affirming Hormone Use Under the Age of 16 eFigure 2. Factors Associated With Obtaining Gender-Affirming Hormone Without Official Prescription eFigure 3. Factors Associated With Positive Feedback on Gender-Affirming Hormone Therapy eTable. Reported Reasons for Regretting Taking Gender-Affirming Hormone Medicines in 21 Chinese Transgender Individuals

Supplement 2. Data Sharing Statement
