# Supplementary material for: Gender Identity Milestones and Hormone Utilization in Transgender Men and Women in China
Source: JAMA Netw Open. 2026 Jan 6;9(1):e2552440. doi: 10.1001/jamanetworkopen.2025.52440 (PMC12776202; doi:10.1001/jamanetworkopen.2025.52440)
Supplement: Supplement 1. — eMethods 1. 2021 Chinese Transgender Health Survey Recruitment eMethods 2. Questions in the 2021 Survey Questionnaire Relevant to this Study eMethods 3. Attention-check Questions in the 2021 Survey eFigure 1. Factors Associated with Gender-Affirming Hormone Use Under the Age of 16 eFigure 2. Factors Associated With Obtaining Gender-Affirming Hormone Without Official Prescription eFigure 3. Factors Associated With Positive Feedback on Gender-Affirming Hormone Therapy eTable. Reported Reasons for Regretting Taking Gender-Affirming Hormone Medicines in 21 Chinese Transgender Individuals [file jamanetwopen-e2552440-s001.pdf]

Supplemental Online Content

Hou J, Pan B, Chen Y, et al. Gender identity milestones and hormone utilization in transgender men and women in China. *JAMA Netw Open.* 2026;9(1):e2552440. doi:10.1001/jamanetworkopen.2025.52440

eMethods 1. 2021 Chinese Transgender Health Survey Recruitment

eMethods 2. Questions in the 2021 Survey Questionnaire Relevant to this Study

eMethods 3. Attention-check Questions in the 2021 Survey

eFigure 1. Factors Associated with Gender-Affirming Hormone Use Under the Age of 16

eFigure 2. Factors Associated With Obtaining Gender-Affirming Hormone Without Official Prescription

eFigure 3. Factors Associated With Positive Feedback on Gender-Affirming Hormone Therapy

eTable. Reported Reasons for Regretting Taking Gender-Affirming Hormone Medicines in 21 Chinese Transgender Individuals

This supplemental material has been provided by the authors to give readers additional information about their work.

© 2026 Hou J et al. *JAMA Network Open.*

eMethods 1. 2021 Chinese Transgender Health Survey Recruitment

The 2021 national Chinese Transgender Health Survey is a national cross-sectional survey conducted from May 6 to December 26, 2021, targeting TGD population in China. The initial recruitment was conducted in our gender clinic in Beijing and through transgender and gender diverse (TGD) community organizations. Our online questionnaire was prepared on the 'Wenjuanxing' platform, and a QR (quick response) code was generated for distribution. We recruited participants by inviting TGD visitors at our clinic and by advertising on TGD organizations' WeChat and Weibo accounts. Those who agreed to participate were given the QR code for online survey entry. Participants were encouraged to share the link on major Chinese social media platforms including WeChat, Weibo and ZhiHu, which are popular in the Chinese TGD community. All questionnaires were anonymous and online informed consent was acquired prior to the study. Participants were informed of the right to withdraw from the study at any time.

eMethods 2. Questions in the 2021 Survey questionnaire relevant to this study

Demographic information

1. How old are you?
2. In which province or region of China were you born and raised?
3. In which province or region of China do you currently reside?
4. What is your educational background?

Measurement of gender identity

1. Which Sex were you assigned at birth?

   – Male – Female – Other (to be filled by participants)

2. If only one item can be chosen, which of the following is a better description of you?

   – Man – Woman – Transgender man – Transgender woman – Non-binary/genderqueer – Cross-dresser

   – Questioning – None of the above

Gender identity milestones related

1. How old were you when you first perceived that your gender identity differed from your sex assigned at birth?
2. How old were you when you first confirmed that your gender identity differed from your sex assigned at birth?
3. How old were you when you first told others that your gender identity differed from your sex assigned at birth?
4. Have you told your family about your gender identity?

   – Yes – No

GAHT utilization related questions

1. Have you ever received gender affirming medical treatment (GAHT) or gender-affirming surgery (GAS)?

© 2026 Hou J et al. *JAMA Network Open*.

– I have taken GAHT – I have taken GAS – I have taken GAHT and GAS – I have taken neither GAHT nor GAS.

2. Are you interested in receiving any form of gender-affirming medical care? – Yes - No.
3. (If yes) Which types of the following means of gender-affirming medical care are you interested in? (select all that apply)
   – GAHT – Secondary sex characteristics modification (e.g. breast, Adam's apple, etc.) – Genital surgery (e.g. vagina, penis, testicles, ovaries, uterus, etc.)
   – Voice modification – Hair removal, transplantation, or extension – Facial surgery – Body contouring procedures (e.g. liposuction, buttock augmentation, etc.)
4. (For those who reported having once taken GAHT) How old were you when you first started GAHT?
5. (For those who reported having once taken GAHT) Are you currently taking GAHT? – Yes – No.
6. Do you obtain hormones with official prescription from a qualified physician? – Yes – No.
7. (If no) What were the main reasons for obtaining hormones without an official prescription? (multiple-choice)
   – Fear of having my transgender identity officially recorded – Unaware of where to access formal medical channels
   – Formal medical procedures are too complicated – Formal medical treatment is too expensive
   – Available medical options do not meet my needs – Dissatisfied with the effects of officially prescribed hormones
   – Other (to be filled by participants)
8. What has been the effect of overall effect of GAHT on your academic or occupational performance?
   – Marked improvement – Moderate improvement
   – No significant change
   – Mild detriment – Significant detriment

eMethods 3. Attention-check questions in the 2021 survey

1. This is an attention check question. The color of space can be green, blue, red, or black, but please select ***green***.
   – Blue – Red – Green – Black
2. This is an attention check question. Please select ***strongly disagree***.
   – Strongly disagree – Disagree – Neutral – Agree – Strongly agree
3. This is an attention check question. Please select the last three words of the following statement: 'Today's weather is ***very not sunny***.
   – Very sunny – Quite sunny – Sunny – Not sunny – Very not sunny

© 2026 Hou J et al. *JAMA Network Open*.

Predictor		Odds ratio (95% CI)	*P* value
**Gender identity**	Transgender men (n = 400)		
	Transgender women (n = 1847)	1.96 (1.32-2.91)	.001
**Early realization of gender incongruence [a]**	No (n = 1236)		
	Yes (n = 1011)	1.27 (0.98-1.64)	.074
**Family disclosure of identity**	No (n = 557)		
	Yes (n = 1690)	1.35 (0.99-1.83)	.055
**Educational background**	Undergraduate or above (n = 1539)		
	Below undergraduate (n = 708)	7.75 (5.92-10.20)	< .001
**Official prescription**	No (n = 1908)		
	Yes (n = 339)	0.93 (0.62-1.39)	.728

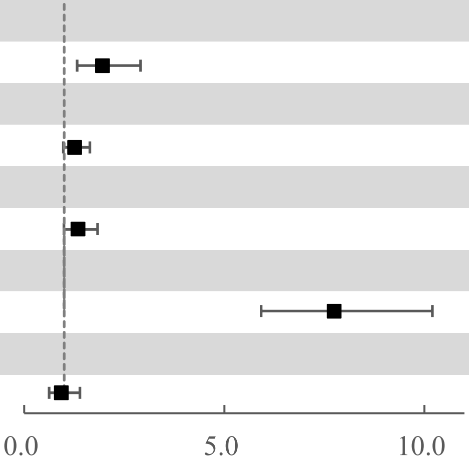

eFigure 1. Factors associated with gender-affirming hormone use under the age of 16.

[a] Early realization of gender incongruence was defined as first perceiving gender incongruence younger than the reported medium age (7 years old for transgender men and 10 years old for transgender women).

© 2026 Hou J et al. *JAMA Network Open*.

Predictor		Odds ratio (95% CI)	*P* value
**Gender identity**	Transgender men (n = 400)		
	Transgender women (n = 1847)	0.54 (0.38-0.76)	< .001
**Early realization of gender incongruence [a]**	No (n = 1236)		
	Yes (n = 1011)	0.92 (0.72-1.16)	.470
**Family disclosure of identity**	No (n = 557)		
	Yes (n = 1690)	0.47 (0.34-0.65)	< .001
**Educational background**	Below undergraduate (n = 708)		
	Undergraduate or above (n = 1539)	0.41 (0.31-0.55)	< .001

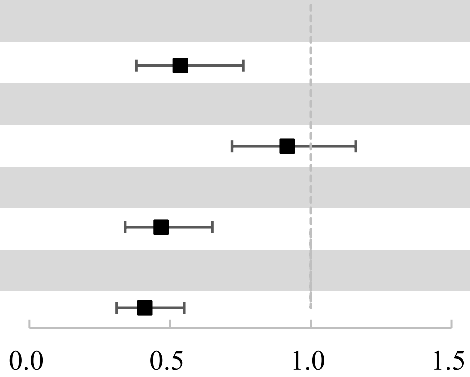

eFigure 2. Factors associated with obtaining gender-affirming hormone without official prescription.

[a] Early realization of gender incongruence was defined as first perceiving gender incongruence younger than the reported medium age (7 years old for transgender men and 10 years old for transgender women).

© 2026 Hou J et al. *JAMA Network Open*.

Predictor		Odds ratio (95% CI)	*P* value
**Gender identity**	Transgender women (n = 1847)		
	Transgender men (n = 400)	3.45 (2.44-4.76)	< .001
**Early realization of gender incongruence [a]**	No (n = 1236)		
	Yes (n = 1011)	0.98 (0.80-1.20)	.849
**Family disclosure of identity**	No (n = 557)		
	Yes (n = 1690)	1.82 (1.47-2.25)	< .001
**Educational background**	Undergraduate or above (n = 1539)		
	Below undergraduate (n = 708)	0.81 (0.65-1.01)	.068
**Official prescription**	No (n = 1908)		
	Yes (n = 339)	1.56 (1.16-2.10)	.004
**Early hormone use [b]**	No (n = 1934)		
	Yes (n = 313)	1.25 (0.92-1.68)	.149

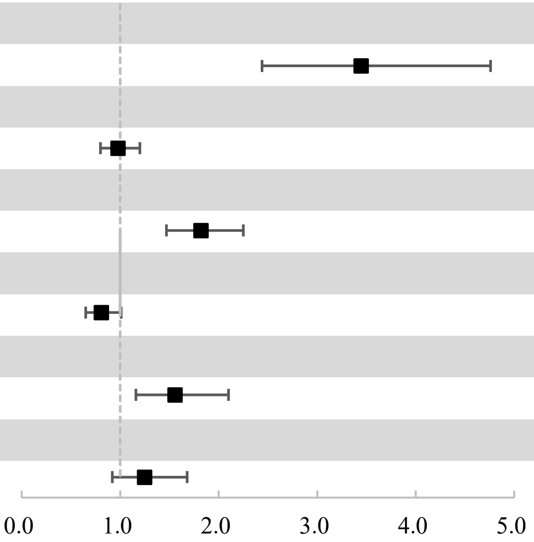

eFigure 3. Factors associated with positive feedback on gender-affirming hormone therapy.

[a] Early realization of gender incongruence was defined as first perceiving gender incongruence younger than the reported medium age (7 years old for transgender men and 10 years old for transgender women).

[b] Early hormone use was defined as first taking hormone medicine under 16 years old.

© 2026 Hou J et al. *JAMA Network Open*.

eTable. Reported reasons for regretting taking gender-affirming hormone medicines in 21 Chinese transgender individuals.

Reported reasons	Number (n)	Percentage (%)
**Not meeting expected body change**	15	83
**Self-reported hormone medicine related adverse events**	10	56
**Not relieving gender anxiety**	9	50
**Cost exceeding financial capacity**	9	50
**Opposition from families**	9	50
**Difficulty to obtain hormones legally**	8	44
**Opposition from friends**	6	33
**Wish for natural reproduction**	4	22

© 2026 Hou J et al. *JAMA Network Open*.
