# Supplementary material for: Gender Identity Milestones and Hormone Utilization in Transgender Men and Women in China
Source: JAMA Netw Open. 2026 Jan 6;9(1):e2552440. doi: 10.1001/jamanetworkopen.2025.52440 (PMC12776202; doi:10.1001/jamanetworkopen.2025.52440)
Supplement: Supplement 2. — Data Sharing Statement [file jamanetwopen-e2552440-s002.pdf]

Data Sharing Statement

Hou. Gender Identity Milestones and Hormone Utilization in Transgender Men and Women in China. *JAMA Netw Open*. Published January 06, 2026.
doi:10.1001/jamanetworkopen.2025.52440

Data
Data available: Yes
Data types: Deidentified participant data
How to access data: yeliumed@bjmu.edu.cn
When available: With publication

Supporting Documents
Document types: None

Additional Information
Who can access the data: Data are not publicly available due to their containing information that could compromise research participant privacy/consent. Data will be made available only to potential collaborators with ethical approval after they submit a research proposal application by contacting the corresponding authors.
Types of analyses: Data will be made available only to potential collaborators with ethical approval after they submit a research proposal application by contacting the corresponding authors.
Mechanisms of data availability: Data will be made available only to potential collaborators with ethical approval after they submit a research proposal application by contacting the corresponding authors.
